# Association of skin-advanced glycation end products, IMT complex, and bone mineral density in patients with type 1 diabetes

**DOI:** 10.3389/fendo.2025.1695436

**Published:** 2025-11-07

**Authors:** Agnieszka Zawada, Dariusz Naskret, Agata Grzelka-Woźniak, Alicja E. Ratajczak-Pawłowska, Anna M. Rychter, Kinga Skoracka, Michał Michalak, Aleksandra Szymczak-Tomczak, Dorota Zozulinska-Ziolkiewicz, Agnieszka Dobrowolska, Iwona Krela-Kaźmierczak

**Affiliations:** 1 Department of Gastroenterology, Dietetics and Internal Diseases, Poznan University of Medical Sciences, Poznan, Poland; 2 Department of Internal Medicine and Diabetology, Poznan University of Medical Sciences, Poznan, Poland; 3 Laboratory of Nutrigenetics, Department of Gastroenterology, Dietetics and Internal Diseases, Poznan University of Medical Sciences, Poznan, Poland; 4 Doctoral School, Poznan University of Medical Sciences, Poznan, Poland; 5 Department of Computer Science and Statistics, Poznan University of Medical Sciences, Poznan, Poland

**Keywords:** advanced glycation end products, bone mineral density, intima media thickness, osteopenia, type 1 diabetes mellitus

## Abstract

**Introduction:**

The accumulation of protein glycation end products, in addition to the direct impact of hyperglycemia, represents one of the most common pathomechanisms involved in the development of osteoporosis in diabetic patients. This study aimed to evaluate the accumulation of advanced glycation end products (AGEs) in the skin and the thickness of the intima/media complex (IMT) in patients with type 1 diabetes in relation to bone mineral density.

**Materials and methods:**

The study comprised a group of 132 individuals, including diabetes mellitus type 1 (DM1) patients. The thickness of the IMT complex was evaluated. Bone mineral density (BMD), T-score, and Z-score were assessed using dual-energy X-ray absorptiometry. Skin AGE assessment was performed by AGE-Reader.

**Results:**

The concentration of AGEs in the skin was significantly higher in patients with DM1 and osteopenia in the femoral neck as compared to individuals with diabetes and normal bone mass, as well as with the control group. The thickness of the IMT complex was significantly greater in subjects with diabetes compared to healthy participants, regardless of osteopenia in the femoral neck and L1-L3.

**Conclusion:**

Patients with DM1 demonstrated lower BMD in L1–L4 and in the femoral neck compared to those in the non-diabetic group. Patients with type 1 diabetes mellitus and osteopenia did not present a statistically significant increase in the thickness of the IMT complex compare to those with DM1 without osteopenia. Individuals with coexisting DM1 and osteopenia in the femoral neck, but not in L1–L4, presented significantly higher values of skin AGEs than participants with diabetes mellitus without bone mineral disorders.

## 1 Introduction

Scientific research proves that increased mortality from cardiovascular diseases is associated with reduced bone mineral density (BMD) and bone fractures (1, 2). The pathomechanism of this phenomenon is not fully understood, although it emphasizes the role of matrix proteins (collagen type 1, proteoglycan, osteopontin, and osteonectin), osteoprotegerin (OPG), hormonal balance by affecting pro-inflammatory cytokines IL-1, IL-6, and TNF-alpha), the activator of the nuclear factor ligand receptor κB, parathyroid hormone phosphates, and vitamins D and K (3). In addition, both osteoporosis and cardiovascular disease are more common in diabetes (4, 5). A general mechanism has also been demonstrated for the occurrence of obesity, insulin resistance, and type 2 diabetes, related to the intensity of inflammation, which—in turn—may increase the risk of osteoporosis and fractures by deteriorating the quality of bone tissue (6). Furthermore, some studies indicated that patients with type 1 diabetes are more likely to develop osteoporosis than individuals with other types of diabetes (4). The severity of osteoporotic changes was also correlated with the severity of the loss of beta cell function and lower C-peptide levels (7). Therefore, it would be relevant to inquire as to the relationship between bone mineral density and cardiovascular diseases in patients with type 1 diabetes.

One of the most common pathomechanisms responsible for the development of osteoporosis in diabetic patients, apart from the direct impact of hyperglycemia, is the accumulation of protein glycation end products. Prolonged hyperglycemia, which occurs in diabetes mellitus, triggers the formation of covalent bonds between the aldehyde group of sugars and the amino group of proteins. The resulting non-enzymatic end products of protein glycation bind to their receptors, leading to the formation of reactive oxygen species and activation of many transcription factors, including the pro-inflammatory nuclear factor kappa-light-chain-enhancer of activated B cells (NFKB) and signaling pathways, such as mitogen-activated protein kinase (MAPK), c-Jun N-terminal kinases (JNKs), and p21RAS (8). Additionally, these conditions also increase the production of many cytokines and growth factors, as well as the expression of intercellular adhesion molecule 1 (ICAM) and vascular cell adhesion molecule (VCAM) (9). Moreover, glycation of collagen IV occurs, which increases its metabolic turnover. In turn, glycation and cross-linking of collagen I and proteoglycan cause their fibers to become stiff and inflexible (10). This may suggest a link between AGE accumulation, incidence of osteoporosis, and severity of atherosclerotic lesions in blood vessels in patients with type 1 diabetes.

Studies conducted on mice have demonstrated the role of the AGE receptor (RAGE) in the metabolism of bone tissue. RAGE knockout mice showed increased bone mass with decreased resorption capacity. RAGE, therefore, enhances bone resorption by osteoclasts (11). In studies conducted on the human population, AGE accumulation has also been widely reported in association with cardiovascular disease, obesity, and other complications of diabetes, such as diabetic retinopathy and nephropathy (12–14).

There is also significant evidence of an association between osteoporosis and peripheral and central vascular calcification, particularly in postmenopausal women. TNF-alpha, produced by macrophages and granulocytes, stimulates the pro-osteoclastic activity of the cell matrix and promotes the formation of osteoclasts and bone resorption (15). Both TNF-alpha and other interleukins play a fundamental role in the formation of atherosclerotic plaque and thus influence the development of cardiovascular diseases (16). Nevertheless, it seems impossible to ignore the role of the complex associated with the nuclear factor activator (RANKL receptor activator of NF-kB, RANK ligand). Notably, RANK ligand, by binding to RANK, increases the calcification of vascular smooth muscle cells directly and increases the production of bone morphogenetic proteins, interleukin 6 (IL-6), and TNF-alpha (17).

The presented study investigated whether skin AGE deposition and thickening of the intima/media complex (IMT) complex are associated with reduced bone mineral density in individuals with type 1 diabetes. It also aimed to evaluate the accumulation of advanced glycation end products (AGEs) in the skin and the thickness of the IMT complex in patients with type 1 diabetes in relation to the bone mineral density.

## 2 Materials and methods

The retrospective study comprised 132 participants and included patients with type 1 diabetes (*n* = 66) aged 39 ± 8 years, as well as the control group of (*n* = 66) healthy individuals aged 37 ± 8 years. The type 1 diabetes group was divided into two subgroups, depending on whether BMD showed a decreased bone mass at the stage of osteopenia or a normal bone mass. The group with osteopenia consisted of 12 patients (*n* = 12), and the group without osteopenia included 54 participants. Osteopenia was diagnosed on the basis of two measurements: DXA (dual-energy X-ray absorptiometry) densitometric assessment of the femoral neck and lumbar spine. The study included patients with type 1 diabetes of confirmed autoimmune etiology, treated with functional insulin therapy or using a personal insulin pump. All patients were Caucasian, and diabetes persisted for over 5 years. All subjects provided informed consent to participate in the study. Individuals who presented with conditions that could affect bone mineral density, such as the presence of a period of diabetes remission, pregnancy, or menopause in women, as well as the presence of chronic diabetic kidney disease with GFR < 60, were excluded from the study. Moreover, patients undergoing chronic treatment with steroids or other drugs affecting bone mineral metabolism, as well as those with previously diagnosed osteopenia or osteoporosis, were also excluded. Finally, individuals with coexisting celiac disease and other absorption disorders were also excluded from the study.

The following tests were performed in the study group: the thickness of the IMT complex was evaluated, and the accumulation of AGEs in the skin was assessed.

### 2.1 Assessment of intima/media complex thickness measurement

Evaluation of the thickness of the IMT complex was conducted in all individuals while performing a standard Doppler examination using a Simens CV 70 apparatus. Automatic analysis of the results of the IMT complex thickness was performed using a device-Carotid Analyzer for Research. The person performing the IMT measurement was not informed whether the patient was in the study group or in the control group.

### 2.2 Assessment of AGE content in the skin

Quantitative assessment of the content of end products of protein glycation in the skin was performed using the AGE-Reader device (DiagnOptics Technologies B.V., The Netherlands, type 214D00102). This device is equipped with an ultraviolet light source in the wavelength range of 300–420 nm. The autofluorescence index (AF) is the quotient of the average light intensity emitted in the 420–600 nm wavelength range to the average light intensity in the 300–420 nm wavelength range. AF is expressed in arbitrary units and multiplied by 100. For each patient, AF was measured three times in a series, and the result was the arithmetic average of these evaluations. The measurement lasted 30 s; it was performed at room temperature on the ventral side of the forearm, approximately 5 cm distal to the elbow. It is essential that the skin be free of tattoos, damage, and cosmetics containing UV filters.

### 2.3 Bone mineral density

Bone mineral density, T-score, and Z-score of the lumbar spine (L1–L4) and femoral neck (FN) were assessed using dual-energy X-ray absorptiometry with Lunar DPX-Plus (Lunar, Inc., Madison, Wisconsin, USA). Osteopenia was diagnosed as a T-score value of −1 to −2.5. Osteoporosis was diagnosed as a T-score value below −2.5. A T score above −1 was considered normal. Individuals with a T-score less than −1 were excluded from the control group, whereas patients with previous bone fractures were excluded from both groups.

### 2.4 Statistical analysis

#### 2.4.1 Sample size

We aimed to detect a medium omnibus effect (Cohen’s f = 0.30) at α = 0.05 and 80% power. Under the assumption of equal group sizes and using the ANOVA approximation for a three-group comparison, the required total sample size is N ≈ 111N (≈ 37 per group). Our resources allowed us to recruit 121 participants.

#### 2.4.2 Sample size adequacy and sensitivity analysis

For the omnibus between-group comparisons we reported effect sizes as Cohen’s f (derived from η² ≈ ϵ² for Kruskal–Wallis). Using an ANOVA approximation to Kruskal–Wallis with α = 0.05, power = 0.80, and unequal group sizes adjusted via the harmonic-mean equivalent N, we obtained a minimal detectable effect of f = 0.319. All significant outcomes in our study exceeded this threshold (the lowest IMT f = 0.415 and the highest AGE f = 0.846). For comparison of two groups, we used as an effect size Cliff’s δ.

The data derived from the interval scale did not meet the assumption of normality (Shapiro–Wilk test). Therefore, comparisons between the studied groups, namely, diabetes mellitus type 1 (DM1) +osteoporosis, DM1 with normal BMD, and the control group, were performed using the non-parametric Kruskal–Wallis test. When significant differences were identified, *post-hoc* Dunn’s tests (with Holm adjustment for multiple testing) were conducted to determine homogeneous groups. The results are presented as medians (Me) and interquartile ranges (Q1–Q3). For the analysis of categorical data, the chi-square test for independence was applied, and in cases where Cochran’s criterion was not met, Fisher’s exact test was used. The results were presented as numbers and relative percentages. All tests were considered significant at *p* < 0.05. The statistical analysis was conducted using the PQStat Software package (2022), PQStat v.1.8.4.164, Poland, www.pqstat.pl.

### 2.5 Ethics committee

The study protocol was approved by the Poznan University of Medical Sciences Bioethics Commission (approval no. 486/21). The study protocol conforms to the ethical guidelines of the 1975 Declaration of Helsinki, as reflected in a prior approval by the institution’s human research committee. All patients gave written informed consent.

## 3 Results

The median age of patients included in the study was on average 34.9 years in the group with T1DM and osteopenia in the femoral neck and 40 years in T1DM without osteopenia in the femoral neck. The median age of patients with type 1 diabetes and osteopenia in L1–L4 was 41.7 years versus 39.9 years in T1DM without osteopenia in L1–L4. Median age in the control group was 38.5 years. The average duration of diabetes was 15 years both in the group with and without osteopenia in the femoral neck, 18 years in the group with osteopenia in L1–L4, and 14.5 years in the T1DM group without osteopenia in the L1–L4 group. The median value of glycated hemoglobin was 8.0% in the group with osteopenia in the femoral neck and T1DM, whereas in the group without osteopenia in the femoral neck it was 7.8%. The median value of glycated hemoglobin was 7.5 in the group with osteopenia in L1–L4 and T1DM; in the group without osteopenia in L1–L4, it was equal to 7.8%. Interestingly, we did not observe osteoporosis in the study group. The characteristics of anthropometric, densitometric, and biochemical parameters in patients with type 1 diabetes and in the control group are presented in **Tables 1** and **2**, depending on the presence of osteopenia in the femoral neck and L1–L4 section. Our study demonstrated that in patients with type 1 diabetes, the incidence of osteopenia was the same in both study sites, and it was found in 12 out of 66 participants (18%). The mean BMI in the osteopenia (FN) group was 23.83 kg/m^2^ and 24.75 kg/m^2^ (L1–L4). However, in the DM1 group without osteopenia (FN), it was 24.70 kg/m^2^.

**Table 1:** Bone mineral density in the femoral neck measured by dual-energy X-ray absorptiometry in patients with type 1 diabetes and the control group.

Variable	GC (*n* = 66)	DM1+ normal BMD (*n* = 54) (FN)	DM1+ OST (*n* = 12) (FN)	*P-*value
	Median value Q1–Q3	Median value Q1–Q3	Median value Q1–Q3	
Age, y	38.50 28.50–43.50	40 32.50–45.10	34.90 (28.95–44.20)	0.47
Sex, female/male, n (%)	45 (68%)/ 21(32%)*	26(48.15)/28(51.85)*	6(50.00)/6 (50)*	0.07
Body weight, kg	68.00 60.50–79.00	76.00 63.00–89.00	69.50 (62.00–80.00)	0.15
BMI, kg/m^2^	23.00 20.80–26.40	24.70 21.55–28.00	23.83 21.75–25.80	0.21
WHR, n	0.84 0.80–0.90	0.89 0.84–0.92	0.89 0.85–0.96	0.28
25(OH)D mg/dl	30.50 22.00–36.00	27.50 21.00–41.00	29.00 19.00–38.50	0.92
BMD (FN), g/cm^2^ *f* = 0.547	**1.10** **1.03–1.19**	**1.06** **1.00–1.12**	**0.89** **0.87–0.90**	**< 0.001** **0.23^a^** **< 0.001^b^** **< 0.001^c^**
T-score (FN) *f* = 0.570	**0.40** **0.00–1.10**	**0.00** **(-0.30)–(0.60)**	**−1.25** **(−1.50)–(1.10)**	**< 0.001** **0.1^a^** **< 0.001^b^** **< 0.001^c^**
Z-score (FN) *f* = 0.522	**0.60** **0.20–1.30**	**0.30** **(-0.10)–(0.90)**	**-0.90** **(-1.10)–(0.70)**	**< 0.001** **0,12^a^** **< 0.001^b^** **< 0.001^c^**
Diabetes duration, y	- -	15.0 7.0–23.0	15.00 10.0–26.0	0.52
DDI, u/kg/day Cliff’s |δ| = 0.462	- -	**0.56** **0.48–0.63**	**0.63** **0.60–0.71**	**< 0.05^c^**
HbA1c, %	- -	7.80 6.80–8.40	8.00 7.40–8.60	0.42^c^
FPG, mg/dl	88.00 84.00–94.00	150.0 116.0–228.0	210.00 84.0–278.0	0.58
Creatinine, μmol/l	69.80 60.90–81.30	0.84 0.73–0.95	0.91 0.79–0.98	0.59

BMD, bone mineral density; CG, control group; DM1, type 1 diabetes; FPG, fasting plasma glucose; FN, femoral neck; L1–L4, Lumbar spine (L1–L4); 25(OH)D, 25−hydroxyvitamin D; WHR, waist to hip ratio.

*f*-Cohen’s *f* effect size.

a. DM1+ normal BMD versus CG.

b. DM1 + OST versus CG.

c. DM1+OST versus DM1+ normal BMD.

Classification according to the occurrence of osteopenia and normal bone mineral density.

Bold values - statistically significant.

**Table 2:** Characteristics of the study group—bone mineral density in L1–L4 measured by dual—energy X-ray absorptiometry in patients with type 1 diabetes and the control group.

Variable	GC (*n* = 66)	DM1 + normal BMD (*n* = 54) (L1–L4)	DM 1+ OST (*n* = 12) (L1–L4)	*P-*value
	Median value Q1–Q3	Median value Q1–Q3	Median value Q1–Q3	
Age, *y*	38.50 28.50–43.50	39.90 32.40–44.80	41.70 33.10–45.70	0.70
Sex, female/male, *n* (%)	45 (68%)/21(32%)*	28 (52.83)/25 (47.17) *	4 (30.77)/9(69.23)*	< 0.05
Body weight, kg	68.00 60.50–79.00	73.00 66.00–88.00	77.00 60.00–88.00	0.23
BMI, kg/m^2^	23.00 20.80–26.40	24.75 21.30–28.15	24.05 22.60–24.95	0.16
WHR, *n*	0.84 0.8–0.9	0.89 0.83–0.93	0.89 0.85–0.92	0.25
25(OH)D, (mg/dl)	30.5 22.0–36.0	28.00 20.00–44.00	26.00 21.00–33.00	0.69
BMD (L1–L4) g/cm^2^ *f* = 0.585	**1.22** **1.17–1.33**	**1.23** **1.17–1.32**	**1.02** **0.98–1.06**	**< 0.001** **1.00^a^** **< 0.001^b^** **< 0.001^c^**
T-score (L1–L4) *f* = 0.571	**0.25** **(−0.30)–(1.25)**	**0.30** **(−0.30)–(0.90)**	**(-1.50)** **(−1.70)–(−1.20)**	**< 0.001** **1,00^a^** **< 0.001^b^** **< 0.001^c^**
Z-score (L1–L4) *f* = 0.504	**0.35** **(−0.20)–(1.00)**	**0.25** **(−0.40)–(0.85)**	**(−1.30)** **(−1.90)–(−1.20)**	**< 0.001** **1.00^a^** **< 0.001^b^** **< 0.001^c^**
Diabetes duration, *y*	- -	14.50 8.00–23.00	18.00 12.0–24.0	0.21
DDI, µ/kg/day	- -	0.56 0.48–0.67	0.60 0.53–0.63	0.36
HbA1c, %	- -	8.00 6.9–8.60	7.5 7.0–8.3	0.49
FPG, mg/dl	88 84.0–94.0	144.00 112.5–224.0	210.0 176.00–278.00	0.72
Creatinine, μmol/l	69.8 60.9–81.3	0.84 0.73–0.95	0.89 0.76–1	0.49

BMD, bone mineral density; CG, control group; DM1, type 1 diabetes; FPG, fasting plasma glucose; OST, osteopenia.

*f*-Cohen’s f effect size.

a. DM1+ normal BMD versus CG.

b. DM1 + OST versus CG.

c. DM1+OST versus DM1+ normal BMD.

Classification according to the occurrence of osteopenia and normal bone mineral density.

Bold values - statistically significant.

The average BMI value in the control group was 23 kg/m^2^. The control group, the type 1 diabetes and osteopenia, as well as the type 1 diabetes without osteopenia group did not differ in the mean vitamin D level and age. The groups with type 1 diabetes and osteopenia, as well as with type 1 diabetes without osteopenia, did not differ in HbA1c values and the duration of diabetes. However, in groups with T1DM and osteopenia in the femoral neck, a significant statistical difference was noted in the daily dose of insulin.

The differences in IMT and AGE complex values in relation to the occurrence of osteopenia of the femoral neck in patients with type 1 diabetes compared with individuals with diabetes without osteopenia and the control group are presented in **Table 3**.

**Table 3:** Intima media thickness and advanced glycation end product skin concentration values in relation to the occurrence of osteopenia in type 1 diabetic patients and control group.

Variable	CG (*n* = 66)		DM1 + normal BMD (*n* = 54) (FN)		DM 1 + OST (*n* = 12) (FN)		*P-*value
	Median value (Q1–Q3)		Median value (Q1–Q3)		Median value (Q1–Q3)		
AGE, *n* *f* = 0.846	1.6	1.4–2.0	2.3	1.9–2.7	3.15	2.65–3.65	< 0.001 < 0.001^a^ < 0.001^b^ < 0.001^c^
IMT, *n* *f* = 0.415	0.54	0.49–0.59	0.62	0.54–0.69	0.63	0.56–0.72	< 0.001 < 0.001^a^ < 0.01^b^ 1.00^c^

AGE, advanced glycation end products; IMT, intima media complex.

*f*-Cohen’s *f* effect size.

a. DM1+ normal BMD versus CG.

b. DM1 + OST versus CG.

c. DM1+OST versus DM1+ normal BMD.

The concentration of AGEs in the skin was significantly higher in patients with type 1 diabetes and osteopenia in the femoral neck compared to subjects with diabetes and normal bone mass and the control group. The thickness of the IMT complex was significantly greater in participants with diabetes compared to healthy individuals, regardless of the presence of osteopenia in the femoral neck.

Moreover, higher AGE values were observed in the skin in patients with type 1 diabetes and osteopenia in the L1–L4 section as compared to patients with type 1 diabetes and normal bone mass and the control group (**Tables 4**, **5**).

**Table 4:** Difference in intima media thickness and advanced glycation end product skin concentration values in relation to the occurrence of osteopenia of the lumbar spine in patients with type 1 diabetes compared to individuals with diabetes without osteopenia and the control group.

Variable	CG (*n* = 66)		DM1 + normal BMD (*n* = 54) (L1–L4)		DM 1 + OST (*n* = 12) (L1–L4)		*P-*value
	Median value (Q1–Q3)		Median value (Q1–Q3)		Median value (Q1–Q3)		
AGE *f* = 0.613	1.6	1.4–2.0	2.5	2.1–2.6	2.3	2.1–3.0	< 0.001 < 0.001^a^ < 0.001^b^ 1.00^c^
IMT *f* = 0.407	0.54	0.49–0.59	0.61	0.58–0.67	0.62	0.54–0.71	0.001 < 0.001^a^ 0.02^b^ 1.00^c^

AGE, advanced glycation end products; IMT, intima media complex.

*f*-Cohen’s *f* effect size.

a. DM1+ normal BMD versus CG.

b. DM1 + OST versus CG.

c. DM1+OST versus DM1+ normal BMD.

**Table 5:** Intima media thickness and advanced glycation end product skin concentration values in relation to the combined occurrence of osteopenia in the femoral head and the L1–L4 spine segment in type 1 diabetic patients and control group.

Variable	CG (*n* = 66)		DM1 + normal BMD (*n* = 45) (FN+ L1–L4)		DM 1 + OST (*n* = 21) (FN+L1–L4)		*P-*value
	Median value (Q1–Q3)		Median value (Q1–Q3)		Median value (Q1–Q3)		
AGE, *n* *f* = 0.742	1.6	1.4–2.0	2.3	1.95–2.7	2.6	2.3–3.2	< 0.001 < 0.001^a^ < 0.001^b^ 0.049^c^
IMT, *n* *f* = 0.483	0.54	0.49–0.59	0.62	0.53–0.71	0.63	0.58–0.68	< 0.001 0.002^a^ 0.0005^b^ 0,99^c^

AGE, advanced glycation end products; IMT, intima media complex.

*f*-Cohen’s *f* effect size.

a. DM1+ normal BMD versus CG.

b. DM1 + OST versus CG.

c. DM1+OST versus DM1+ normal BMD.

## 4 Discussion

The occurrence of osteoporosis and osteopenia constitutes a serious complication for the elderly. However, it may develop at an earlier age when other diseases, such as type 1 diabetes, are also present. Studies have shown that patients with type 1 diabetes are at greater risk of developing bone mineral disorders than the general population (18). The pathomechanism of this phenomenon is complex; however, it seems that end products of protein glycation play a crucial role in this process. According to the study by Ge et al., patients with osteoporosis demonstrated higher serum AGE levels as compared to healthy subjects, and serum AGE levels were negatively correlated with body mass density (BMD) (19, 20). The concentration of one of the main products of protein glycation, pentosidine, is significantly increased in patients with type 1 diabetes. Several studies have also confirmed that pentosidine accumulation is associated with a higher risk of osteoporotic bone fractures (21–23). The clinical study by Neumann et al. found an association between the accumulation of AGEs and fractures in patients with DM1, once other risk factors, such as age and smoking, were accounted for (24). Other studies have also confirmed the association of AGE accumulation with poorer bone quality (25). In addition, long-term type 1 diabetes leads to the accumulation of AGEs and bone tissue deficit (26). AGE-enhanced apoptosis of osteoblasts through activation of the receptor for advanced glycation end products (RAGE) may be responsible for this phenomenon (27). In addition, AGEs interfere with bone turnover and deteriorate bone quality through changes in the organic matrix (28, 29). They affect both collagen and non-collagen proteins. Our study showed a statistically significant difference between AGE values in the skin in subjects with type 1 diabetes, depending on the presence of osteopenia. This was also confirmed in the larger group resulting from the combination of patients with osteopenia in both areas of assessment (femoral head and L1–L4 spine segment together). Thereby, the mere demonstration of such a difference at a preliminary and reversible stage of bone disorders, such as osteopenia, may be of considerable clinical significance. Performing a minimally invasive assessment of AGEs in the skin using autofluorescence may help to identify individuals who should be screened for osteopenia and osteoporosis. The identification of such a patient group may result in the implementation of early prevention and prevent the progression of osteopenia into osteoporosis.

The thickness of the IMT complex has been a well-established indicator of cardiovascular risk for a number of years, both in the general population and in individuals suffering from type 1 diabetes (30, 31). Various scientific sources indicate that reduced bone mass may correlate with early markers of cardiovascular risk (32). The prospective Young Finns Study (YFS), which evaluated cardiovascular risk factors from childhood to adulthood in a population of healthy young Finnish adults, revealed an association between early cardiovascular risk markers as assessed by IMT complex thickness and reduced bone mineral density (33). However, no such studies have been conducted on smaller populations, such as patients with type 1 diabetes, where the risk of both diseases is elevated. In contrast, studies conducted on other populations of individuals with autoimmune diseases have shown a similar relationship. It has been reported that in a group of 50 women with rheumatoid arthritis, 56% showed early arteriosclerotic lesions in the brachiocephalic arteries, whereas 62% developed osteoporosis and osteopenia. An inverse relationship between the thickness of the intima-media complex of the brachiocephalic arteries and bone mineral density in the proximal femur and lumbar spine was also confirmed (34). Our study demonstrated the thickening of the intima media complex in participants with type 1 diabetes and osteopenia and thus provides new insights into the coexistence of bone microarchitectural abnormalities and cardiovascular diseases in patients with type 1 diabetes. Nonetheless, we did not observe a statistically significant increase in the thickness of the intima-media complex in individuals with early stage bone microstructural abnormalities, such as osteopenia, compared to the control group. Furthermore, despite the lack of statistical differences, the thickness of the IMT complex was greater in the group with diabetes and osteopenia than in the group with diabetes alone. The lack of statistical differences is potentially associated with the early stage of bone mineralization disorder, that is, osteopenia.

To summarize, impaired bone mineral metabolism is possibly associated with chronic hyperglycemia. This condition, in turn, affects the process of atherogenesis by increasing the amount of protein glycation end products in the body. The onset of early and reversible bone loss, such as osteopenia, likely results in an increased accumulation of AGEs in the skin, leading to the appearance of early markers of atherogenesis.

### 4.1 Conclusions

1. Patients with type 1 diabetes demonstrated lower bone mineral density in both the lumbar spine and the femoral neck compared to those in the non-diabetic group.
2. Patients with type 1 diabetes mellitus and osteopenia, as indicated by the assessment of mineral density of the femoral neck and the lumbar segment of the spine, did not present a statistically significant increase in the thickness of the intima media complex compared to individuals with type 1 diabetes mellitus without osteopenia.
3. Patients with coexisting type 1 diabetes mellitus and osteopenia in the femoral neck presented slightly higher values of skin AGEs than participants with diabetes mellitus without bone mineral disorders.

### 4.2 The limitations of the study

The study’s limitations included a relatively small research group, a small subgroup with osteopenia, and restrictions related to the AGE measurement technique in the skin using the AGE Reader device.

This study is retrospective, which entails potential selection bias and limited control over confounding variables. The osteopenia subgroup was small, and the groups were unequal in size; although we used the Kruskal–Wallis test and conducted a sensitivity analysis based on an ANOVA approximation, this may limit the detectability of very small between-group differences. At the same time, for all statistically significant comparisons the observed effect sizes (Cohen’s f) exceeded the 80% power threshold determined for our sample sizes, which supports the robustness of the main findings.

## Data Availability

The raw data supporting the conclusions of this article will be made available by the authors, without undue reservation.
